# Supplementary material for: Pick and place process for uniform shrinking of 3D printed micro- and nano-architected materials
Source: Nat Commun. 2023 Sep 21;14:5876. doi: 10.1038/s41467-023-41535-9 (PMC10514194; doi:10.1038/s41467-023-41535-9)
Supplement: Supplementary file 1 — Supplementary Information [file 41467_2023_41535_MOESM1_ESM.pdf]

Supplementary Information

Pick and Place Process for Uniform Shrinking of 3D Printed Micro- and Nano-Architected Materials

Tomohiro Mori[1,2], Hao Wang[1,3,4*], Wang Zhang[1], Chern Chia Ser[1], Deepshikha Arora[1], Cheng-Feng Pan[1,5], Hao Li[1], Jiabin Niu[1], M. A. Rahman[1], Takeshi Mori[2], Hideyuki Koishi[2], Joel K. W. Yang[1*]*

Affiliations

[1]Engineering Product Development, Singapore University of Technology and Design, Singapore, 487372, Singapore

[2]Industrial Technology Center of Wakayama Prefecture, Wakayama, 6496261, Japan

[3]College of Mechanical and Vehicle Engineering, Hunan University, Changsha, 410082, China

[4]Greater Bay Area Institute for Innovation, Hunan University, Guangzhou, 511300, China

[5]Department of Electrical and Computer Engineering, National University of Singapore, Singapore, 117576, Singapore

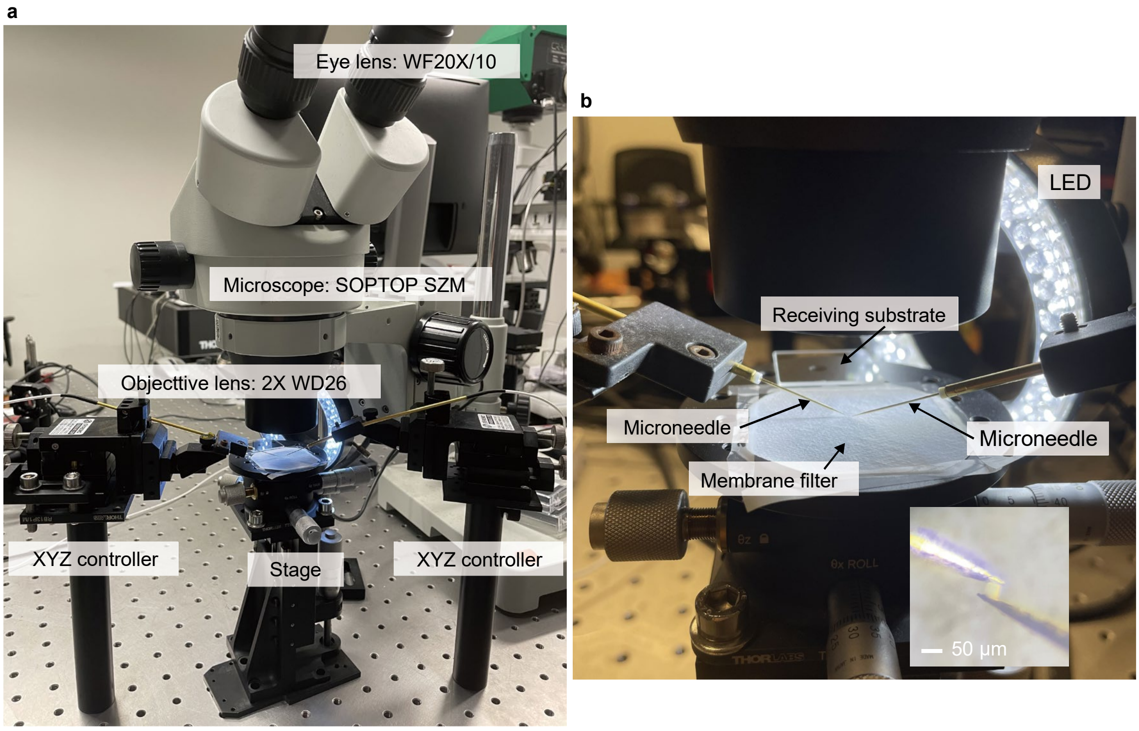

Supplementary Fig. 1 | Custom-made microneedle pickup system. a Whole photograph of the stage **b** Close-up photograph of the stage. The inset optical image shows the top view of the transfer of the 3D woodpile structure using the two microneedles.

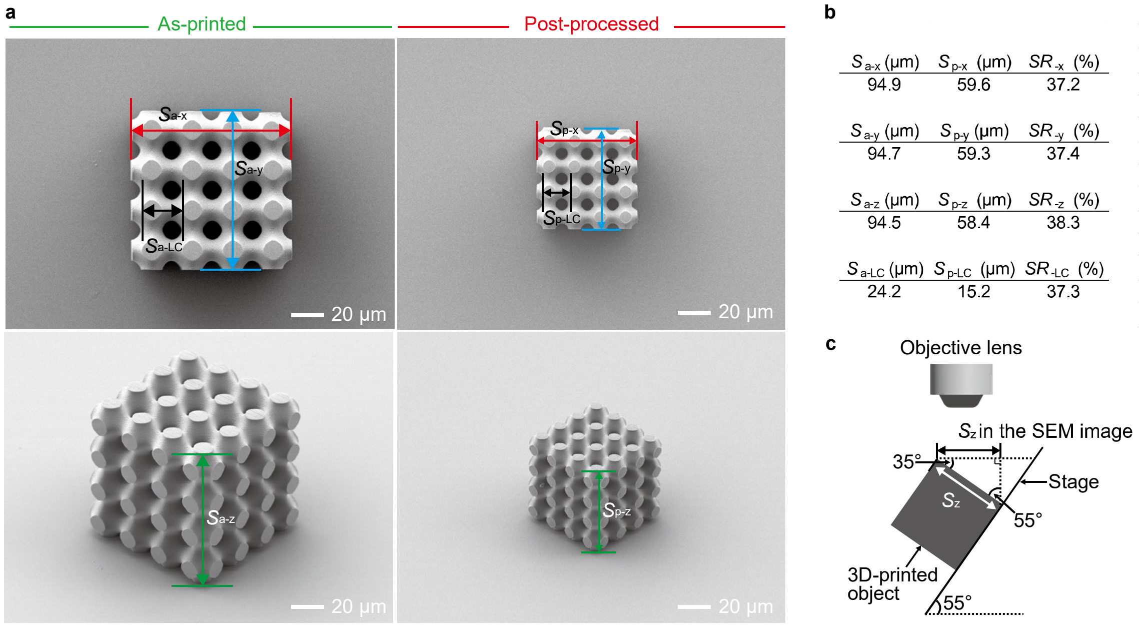

Supplementary Fig. 2 | Measurement of the shrinkage rate (*SR*) in different directions. The 3D simple cubic model was heated up to and maintained at 450 °C for 5 min on the receiving substrate. **a** SEM images of the as-printed and post-processed simple 3D cubic models. The bottom images show side views of the models tilted by 55°. **b** *SR* in each direction and the corresponding lattice constant. **c** Schematic of the calculation of the actual length of $S_{\mathrm{a-z}}$ and $S_{\mathrm{p-z}}$ (S_{z} *in the* SEM image/sin 55°).

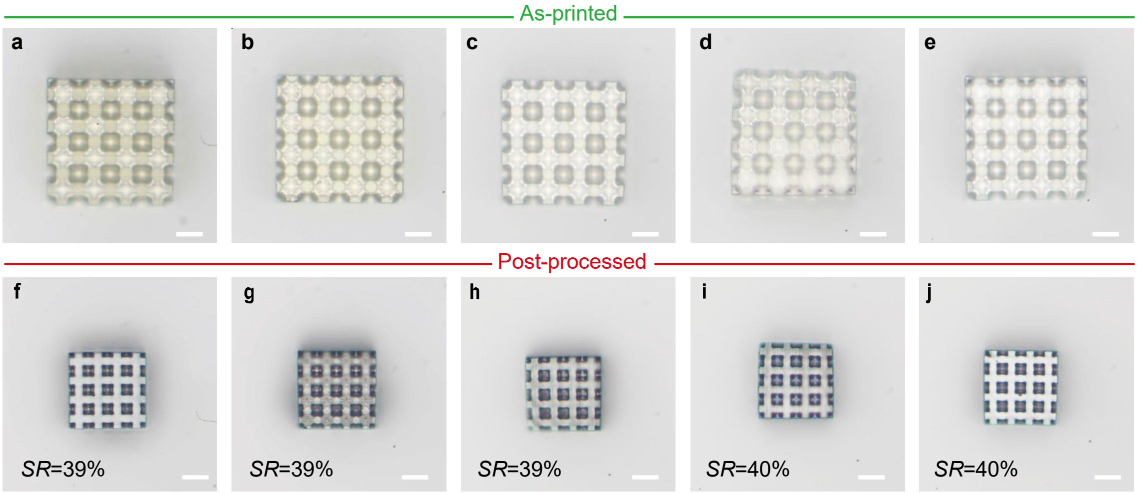

Supplementary Fig. 3 | Bright-field reflective optical images of the 3D printed structures with different faces mounted on the receiving substrate. a–e The as-printed 3D large woodpiles and **f–j** corresponding post-processed 3D large woodpiles. The *SR* of each structure was inserted in the corresponding image. The 3D large woodpiles were heated up to and maintained at 450 °C for 5 min. Scale bars = 20 µm.

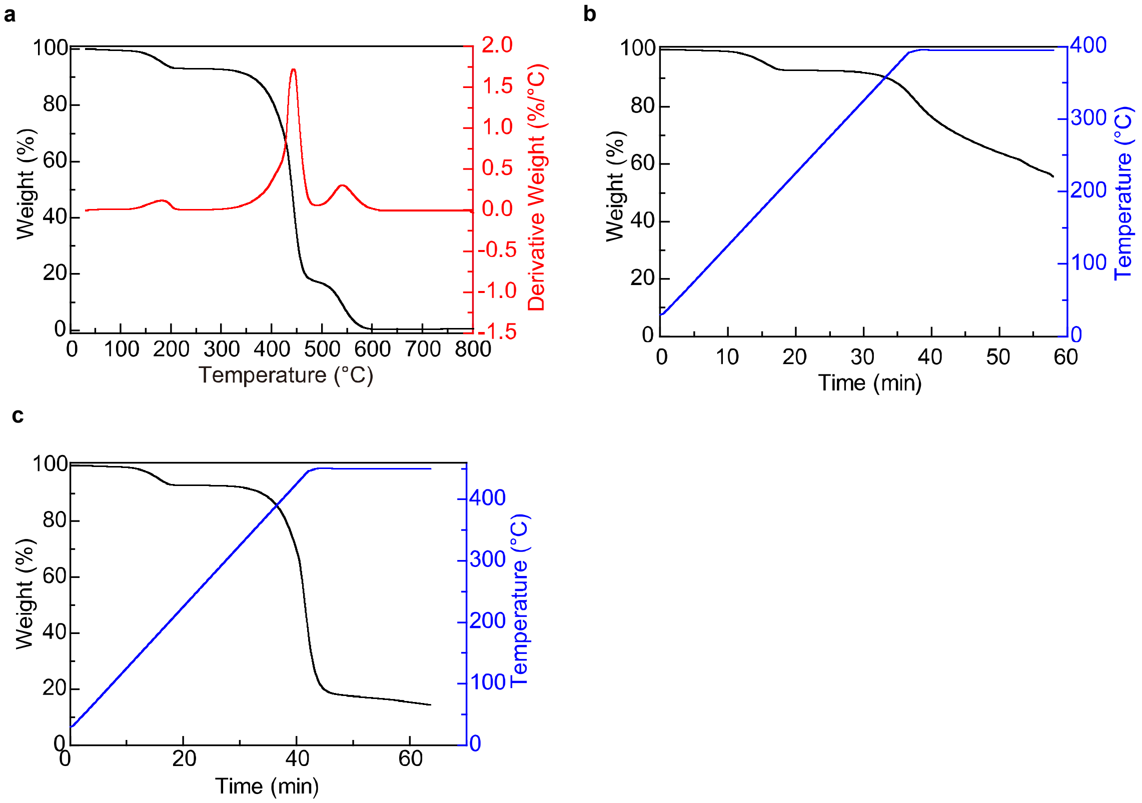

Supplementary Fig. 4 | Results of the thermogravimetric analysis of IP-Dip2 in air. **a** Heating from room temperature (RT) to 800 °C at a rate of 10 °C/min. **b** Heating from RT to 395 °C at a rate of 10 °C/min and maintained at 395 °C for 20 min. **c** Heating from RT to 450 °C at a rate of 10 °C/min and maintained at 450 °C for 20 min. Solid samples of IP-Dip2 were cast on the fused silica substrate and then UV-irradiated at 405 nm (MX-150, Dymax) for 3 min at 75% maximum power.

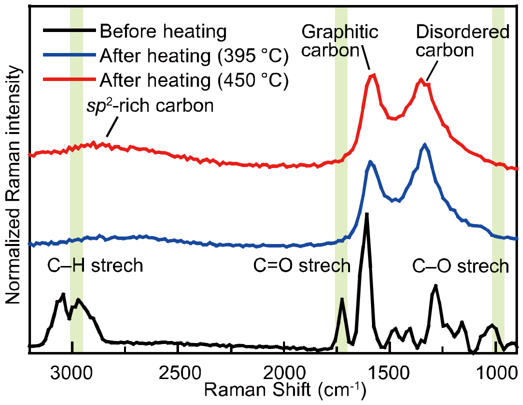

Supplementary Fig. 5 | Raman spectrum of the IP-Dip2 photoresist, which consists of 60–80% 2-(hydroxymethyl)-2-[[(1-oxoallyl)oxy]methyl]-1,3-propanediyl diacrylate (CAS No. 3524-68-3, common name: pentaerythritol triacrylate).

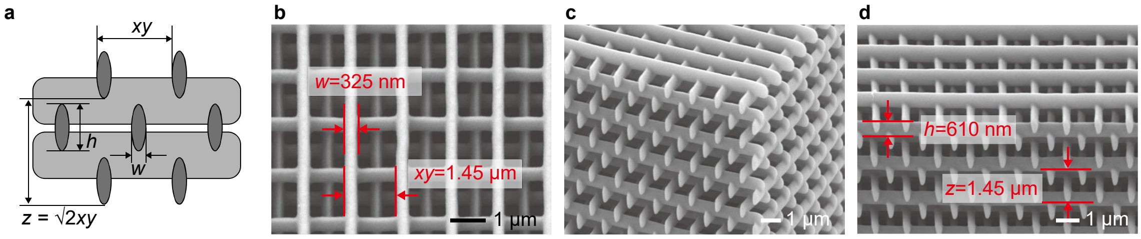

Supplementary Fig. 6 | As-printed woodpile structure. a Schematic showing one unit of the side view. *xy* and *z* represent the lateral and axial lattice distances, respectively. **b** Top view and **c, d** 45°-tilted side view of the SEM images. Writing speed = 9 mm/s, laser power = 24 mW, *xy* (nominal) = 1.65 µm. *w* and *xy* were measured from top view. *h* and *z* were calculated from the height of nanorod and lattice distance at 45°-tilted side view, that is, *h* = 863 nm (610 $nm/sin45°$) and *z* = 2.05 µm (1.45 $\mu m/sin45°$).

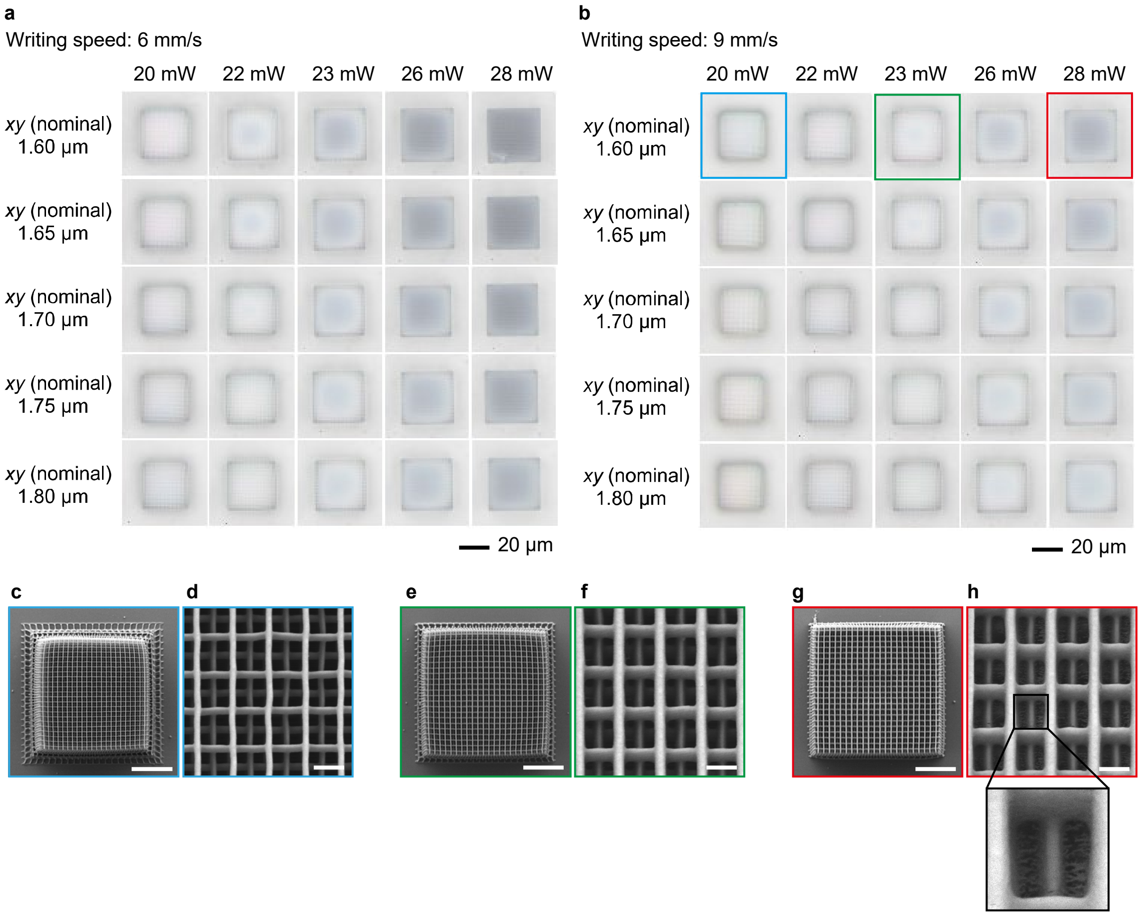

Supplementary Fig. 7 | As-printed woodpile structures prepared under different conditions. a Writing speed = 6 mm/s. **b** Writing speed = 9 mm/s. **c–h** Top view of SEM images corresponding to laser power = 20 mW (blue), 23 mW (green) and 28 mW (red) (*xy* (nominal) = 1.6 μm, writing speed = 9 mm/s). Scale bars in **c, e, g** = 10 μm, scale bars in **d, f, g** = 1 μm.

Note for Supplementary Fig. 7[1-4]

When the exposure power of the laser was reduced or the writing speed was increased, the mechanical stability worsened because of the narrow and weak woodpile lines (Supplementary Fig. 7d). Hence, a large shrinkage of the as-printed woodpile structure occurred after development (Supplementary Fig. 7c) and the post-processed structure collapsed after heating. Meanwhile, when the exposure power of the laser was increased or the writing speed was reduced, the nanoweb formation was generated between the woodpile lines, and the mechanical stability was improved (Supplementary Fig. 7g, h). Therefore, we optimized and selected the conditions for heat shrinking (writing speed = 9 mm/s, laser power = 24 mW, *xy* (nominal) = 1.65 μm).

Supplementary Note 1

Proposed mechanism based on van der Waals (VDW) forces

After remounting 3D printed objects onto a receiving substrate, there is no chemical bond between the 3D printed object and the receiving substrate; they are connected by VDW forces. The expression of VDW forces per unit area between two surfaces is

$$F = -A/12\pi D^3, \qquad (1)$$

where A is the Hamaker constant and D is the distance between the two surfaces.[5] The VDW forces between the 3D printed objects and substrate are less than those of the directly printed structure with perfect contact on the substrate for two reasons: 1) the distance D is increased and 2) the contact area is decreased. Therefore, the total VDW forces, which interfere with gliding on the substrate, decrease, and it becomes easier for the structure to glide.

Supplementary Note 2

Composition of BGL-GZ-83 for anti-sticking layer[6]

1. Methyl nonafluoro butyl ether
   CAS: 163702-07-6, Quantity: 20-80%
2. Methyl nonafluoro isobutyl ether
   CAS: 163702-08-7, Quantity: 20-80%

Information on basic physical and chemical properties:
Physical state: Liquid
Vapour pressure: 26,931 Pa [25 °C]
Viscosity: 0.001 Pa-s [@ 23 °C]
Density: 1.5 g/ml

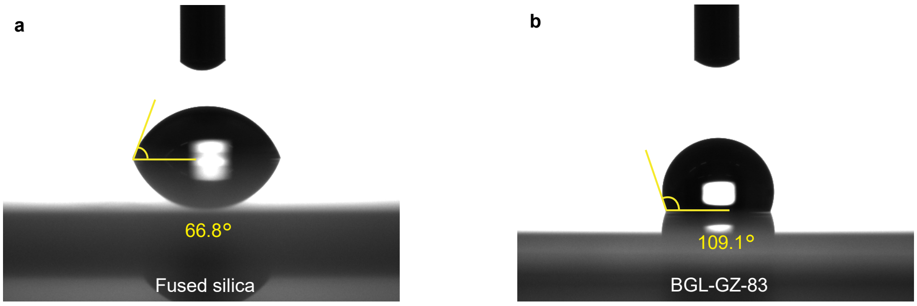

Supplementary Fig. 8 | Optical images of contact angles (water) with the receiving substrate for **a** fused silica substrate and **b** BGL-GZ-83 coated fused silica substrate.

Supplementary Note 3

Measurement of the adhesion force

The adhesion force was measured using a surface-force measurement system (ENT-5X, ELIONIX Inc.). The surface force can be considered to represent the strength of the physical adhesion between two solid materials, unlike the water contact angle measurements employed in the chemical approach[7-9]. As illustrated in Supplementary Fig. 9a, the measurement procedure is as follows. (i) The measurement probe (dimethylpolysiloxane-coated spherical glass probe) with a spring and sample (substrates in our case) are brought near each other. (ii) When the attractive force between the probe and sample exceeds the spring force of the probe, the probe is attracted by the sample, and the two surfaces come into contact. (iii) The probe is pulled off from the sample using an electromagnetic force. (iv) The displacement and pull-off force of the probe are measured with ultra-high resolution (0.03 nN and 0.3 pm). The pull-off force is the force that exceeds the adhesion force between the probe and sample surface. The receiving substrate with an anti-sticking layer exhibited a lower adhesion force (−142.5 μN ± 9.9 μN) than the fused silica substrate without any coating layer (−327.5 μN ± 6.9 μN) (Supplementary Fig. 9b). Here, the absolute value represents the strength of the adhesion force because an attractive force is defined as a negative value. Because the adhesion force of the anti-sticking layer (BGL-GZ-83)-coated fused silica substrate is lower than that of the pure fused silica substrate, the two solid-state materials are relatively easy to separate. These findings support the water contact angle and surface free energy measurement results.

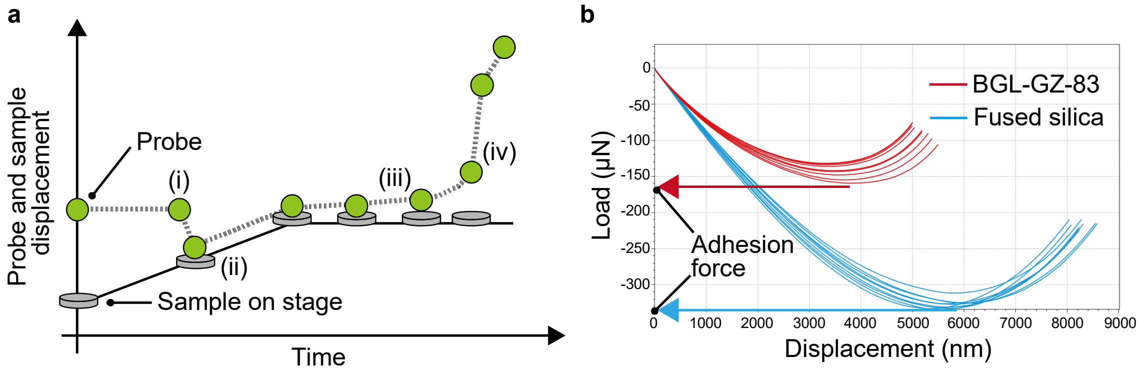

Supplementary Fig. 9 | a Schematic of the adhesion force measurement. **b** Adhesion forces of the anti-sticking layer (BGL-GZ-83)-coated and pure fused silica substrates. The largest absolute value of the load before the displacement jump was determined as the adhesion force.

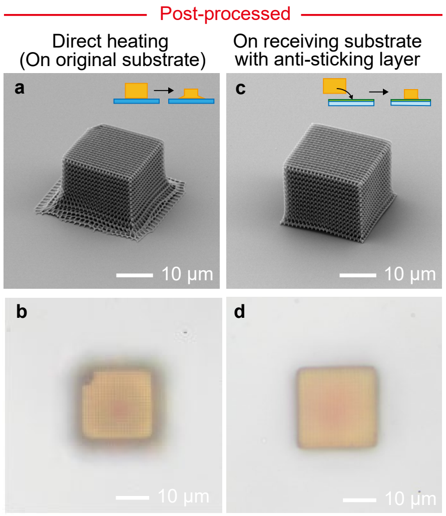

Supplementary Fig. 10 | Comparison of the post-processed woodpiles with and without the pick-and-place process. a, b SEM and bright-field reflective optical images of the post-processed woodpile obtained using conventional direct heating. **c, d** Corresponding images of the post-processed woodpile obtained using the proposed pick-and-place process.

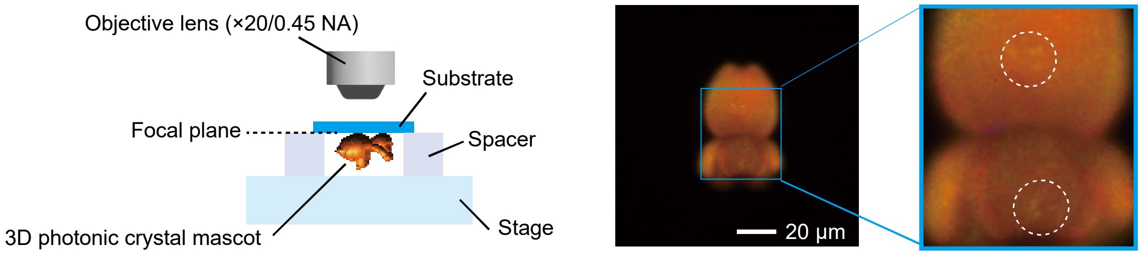

Supplementary Fig. 11 | Adhesion parts between the 3D mascot and substrate after heating. Dark-field reflectance optical images of post-processed 3D mascots were focused on the focal line, as shown in the schematic. The dashed white circles represent the adhesion parts.

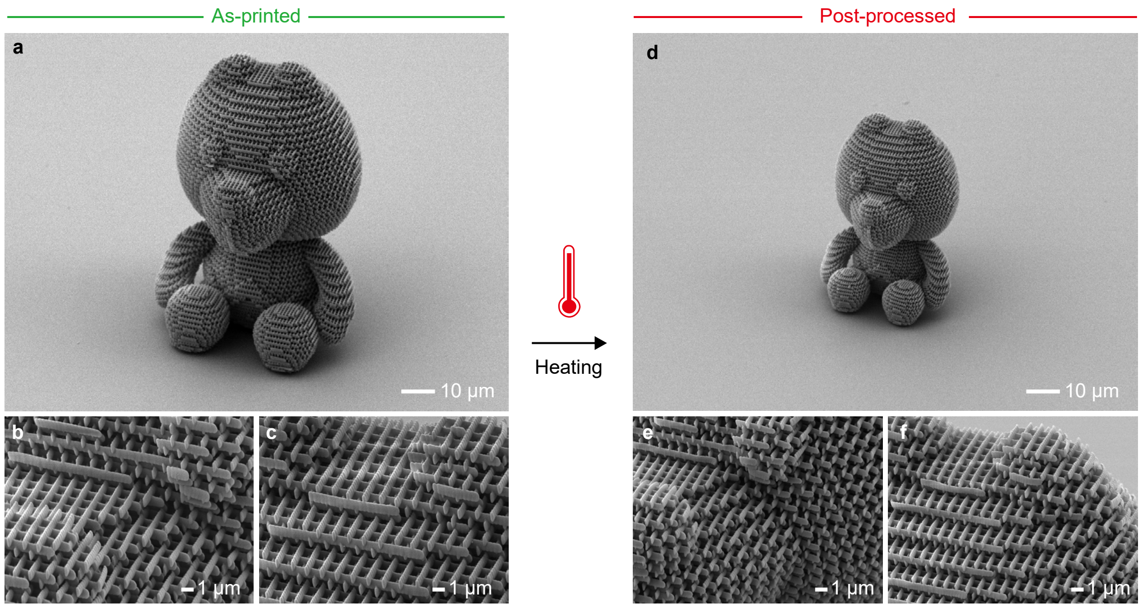

Supplementary Fig. 12 | Uniform shrinkage of printed the 3D photonic crystal mascot with nanoscale features. a-c side view SEM images of the as-printed 3D mascot. **d-f** side view SEM images of the post-processed 3D mascot.

Supplementary References

1. Saha, S. K., Divin, C., Cuadra, J. A. & Panas, R. M. Effect of Proximity of Features on the Damage Threshold During Submicron Additive Manufacturing via Two-Photon Polymerization. *J. Micro Nano Manuf.* **5**, 031002 (2017).
2. Wang, S. et al. Sub-10-nm suspended nano-web formation by direct laser writing. *Nano Futures* **2**, 025006 (2018).
3. Liu, Y. et al. Structural color three-dimensional printing by shrinking photonic crystals. *Nat. Commun.* **10**, 4340 (2019).
4. Wang, H. et al. Toward Near-Perfect Diffractive Optical Elements *via* Nanoscale 3D Printing. *ACS Nano* **14**, 10452–10461 (2020).
5. Israelachvili, J. N. *Intermolecular and Surface Forces* (Academic, 2011).
6. Profactor GmbH ANTI STICKING LAYER BGL-GZ-83. https://www.profactor.at/en/solutions/coatings/.
7. Taniguchi, J., Hasegawa, M., Amemiya, H. & Kobayashi, H. Surface force measurement of ultraviolet nanoimprint lithography materials. *Jpn. J. Appl. Phys.* **55**, 028001 (2016).
8. Kato, T. et al. Effect of roughness on surface force distributions measured by newly developed surface force apparatus with ultra-high accuracy. *Proc. Inst. Mech. Eng. J* **230**, 1336–1344 (2016).
9. Kishimoto, R. et al. Influence of Withdrawal Speed on Adhesion Force. *Tribol. Online* **15**, 60–67 (2020).
